# Changes in the Fermentation and Bacterial Community by Artificial Saliva pH in RUSITEC System

**DOI:** 10.3389/fnut.2021.760316

**Published:** 2021-11-16

**Authors:** Tongqing Guo, Tao Guo, Yurong Cao, Long Guo, Fei Li, Fadi Li, Guo Yang

**Affiliations:** ^1^State Key Laboratory of Grassland Agro-Ecosystems, Key Laboratory of Grassland Livestock Industry Innovation, Ministry of Agriculture and Rural Affairs, Engineering Research Center of Grassland Industry, Ministry of Education, College of Pastoral Agriculture Science and Technology, Lanzhou University, Lanzhou, China; ^2^Gaolan Ecological and Agricultural Integrated Experiment Station, Northwest Institute of Ecological Environment and Resources, Chinese Academy of Sciences, Lanzhou, China

**Keywords:** artificial saliva, rumen bacteria, rumen pH, *in vitro*, ruminant

## Abstract

The purpose of the study was to assess the artificial saliva (AS) pH on ruminal fermentation and rumen bacteria community in the rumen simulation technique (RUSITEC) system. The experiment was performed in two treatments (low AS pH vs. high AS pH) with four replicates. The low AS pH was sustained by altering the composition of the AS (NaHCO_3_ from 9.8 to 1.96 g/L, Na_2_HPO_4_ from 9.3 to 1.86 g/L) according to McDougall's method. The diets were supplemented with 16 g basic diets with forage to the concentrate ratio of 50:50. The experiments were conducted over 13-day incubation periods, with 9 days adaption and 4 days sample collection. The results showed low AS pH decreased dry matter (DM) degradability (64.37 vs. 58.67%), organic matter (OM) degradability (64.38 vs. 59.32%), neutral detergent fiber (NDF) degradability (46.87 vs. 39.94%), acid detergent fiber (ADF) degradability (38.16 vs. 31.13%), and crude protein (CP) degradability (70.33 vs. 62.99%), respectively. Compared with the high AS pH, the low AS pH increased the proportion of butyrate (*P* = 0.008) and decreased the proportion of propionate (*P* < 0.001). At the bacteria community, the low AS pH increased the abundances of *Spirochaetes* (*P* = 0.001) and *Synergistetes* (*P* = 0.004) and decreased the *Verrucomicrobia* abundance (*P* = 0.004) in solid-associated bacteria. At the genus level, the low AS pH increased the abundance of *Lactobacillus* (*P* = 0.050) and decreased the abundance of *Schwartzia* (*P* = 0.002) in solid-associated bacteria. The abundances of *Prevotellaceae_YAB2003_group* (*P* = 0.040), *Schwartzia* (*P* = 0.002), and *Ruminobacter* (*P* = 0.043) were lower in the low AS pH group compared with the high AS pH group in liquid-associated bacteria. Low AS pH decreased the number of *Ruminococcus albus, Ruminococcus flavefaciens, Fibrobacter succinogenes* (*P* < 0.001) both in the solid- and liquid-associated bacteria, respectively. The results of the present study included three groups of bacteria communities according to the different sensitives to rumen pH: the abundances of *Lactobacillus, Succinivibrio, and Prevotella_7* are increased with decreasing AS pH; the amounts of *R. albus, R. flavefaciens, F. succinogenes* as well as the abundances of *Schwartzia* and *Ruminobacter* decreased with the reducing AS pH; the abundances of *Selenomonas_1, Rikenellaceae_RC9_gut_group*, and *Succiniclasticum* were not affected by the AS pH in RUSTITEC.

## Introduction

A high grain-based diet has been a common strategy to improve animal performance in ruminant production. However, the fermentable carbohydrate diets can lead to the accumulation of organic acids in the rumen, which results in the reduction of ruminal pH, and increases the risk of subacute rumen acidosis (SARA) (1, 2). SARA was described as the daily average rumen pH between 6.25 and 5.5 (3). The main SARA model was obtained by increasing the dietary proportions of grain or decreasing physically effective fiber (peNDF) content (4, 5). The SARA induction approaches have a different impact on the rumen fermentation and bacterial community because of the different substrates (6). The low dietary peNDF induced SARA usually increased the feed intake of dairy cows (7), and the increased feed intake resulted in increasing the production of volatile fatty acids (VFA) and decreased pH (8). Therefore, the low peNDF induced SARA needs to avoid the impact of different feed intake between the treatments. Decreasing the peNDF intake for ruminants could reduce the chewing time and the amount of saliva secretion (9). The *in vitro* SARA model that induced in the rumen simulation technique (RUSITEC) system usually by decreasing the buffer capacity of artificial saliva (AS) (10, 11), which could simulate the low peNDF induced SARA. In addition, the RUSITEC system was designed to ensure the identical substrate intake and rumen passage rate during the fermentation that avoids the disturbance of different feed intake and rumen content passage rate of *in vivo* when the ruminants received different dietary peNDF. Orton et al. (10) decreased the buffer capacity of AS (NaCl from 28 up to 118.5 mmol/L and NaCO_3_ from 97.0 to 20 mmol/L) decreased pH from 7.0 to 6.0 in the RUSITEC system.

The ruminal pH plays an important role in affecting the communities of rumen bacteria. Li et al. (8) found the low-peNDF diet induced SARA increased the numbers of *Fibrobacter succinogenes* and *Ruminococcus flavefaciens* for the dairy goats. The increased feed intake and cellulolytic bacteria were due to the more substrates or particulate surfaces available for these bacteria attachment and proliferation (12). The solid-associated bacteria attached to the feed particles play a key role in fiber digestion, while the liquid-associated bacteria have significant functions in the metabolism of soluble nutrients (13, 14). There is a difference in the bacteria community between the solid and liquid fractions. The rumen bacteria are influenced by the combination of substrate, physical structure, and pH environment. Li et al. (15) demonstrated that the three groups of bacteria communities change under grain-induced SARA: pH-sensitive but substrate insensitive bacteria, pH-insensitive but substrate sensitive bacteria, and bacteria that are both pH and substrate sensitive. However, it is difficult to design and execute experiments *in vivo* to test this hypothesis. The RUSITEC system is an optional tool *in vitro* model to simulate the rumen microbial fermentation and could strictly control the effects of substrate and pH independently (10).

Therefore, we hypothesized that the low AS pH would alter the rumen bacteria community, which also lead to the variation of the rumen fermentation. The objectives of this study were to determine the effects of AS pH on the nutrients digestion, rumen fermentation, and ruminal bacteria community.

## Materials and Methods

All the procedures involving animals were carried out in accordance with the Biological Studies Animal Care and Use Committee of Gansu Province, China (2005–2012).

### Equipment, Animals, and Procedures

The study was conducted using RUSITEC (Sanshin, Tokyo, Japan) as described by Kajikawa et al. (16). The RUSITEC system contained eight fermenters with a volume of 800 ml each per tank. The inoculum used in the fermenters was obtained from four ruminal fistulated *Hu* lambs fed two equivalent meals at 07:00 and 19:00 daily in the form of totally mixed ration (TMR) pellets with forage to concentrate of 80:20. The rumen contents were collected through the ruminal fistula before the morning feed and separated into liquid and solid fractions by four layers of cheesecloth. The squeezed solid inoculum (70 g wet weight) was enclosed in a nylon bag (7 × 13 cm, pore size: 100 μm). On the 1st day during fermentation, 400 ml of liquid inoculum was distributed to each fermenter under CO_2_ flux after mixing with an equal volume of AS, and two bags were placed in the fermenter, one with feed and the other with solid inoculum. After 24 h, the bag with the inoculum was replaced by a new bag with the feed. Subsequently, the bag that included the feed incubated 48 h was replaced by a new feed bag. A continuous infusion of AS at a rate of 600 ml/day was maintained in each fermenter. The fermenters were kept in a water bath at 39°C and slowly moved up and down by an electric motor (five times per minute).

### Experimental Diets

The fermenters were randomly assigned to the two treatments with four replicates of each treatment. The treatment included high AS pH (pH 7.0) or low AS pH (pH 6.0) according to McDougall's method (17) (Table 1). The low AS pH was sustained by decreasing the AS buffer capacity (NaHCO_3_ from 9.8 to 1.96 g/L and Na_2_HPO_4_ from 9.3 to 1.86 g/L). The pH of all the fermenters was recorded at 07:30, 15:30, and 23:30 daily throughout the experiment periods. The diets were supplemented with 16 g basic diets with forage to the concentrate ratio of 50:50 (Table 2). The diets were ground through a 2 mm sieve. The experiment was conducted for 13-day incubation periods, with 9 days adaption and 4 days sample collection.

**Table 1:** The composition of the infused buffer^a^.

	**High AS pH**	**Low AS pH**
NaHCO_3_	9.8 g/L	1.96 g/L
Na_2_HPO_4_	9.3 g/L	1.86 g/L
NaCl	0.47 g/L	0.47 g/L
KCl	0.57 g/L	0.57 g/L
MgSO_4_·7H_2_O	0.12 g/L	0.12 g/L
CaCl_2_·2H_2_O	0.045 g/L	0.045 g/L

a *The infused buffer was referenced to McDougall's method (17)*.

**Table 2:** The dietary ingredients and nutrient composition (% dry matter [DM]).

**Ingredients**	**Contents**
Alfalfa hay, %	40.60
Corn straw, %	9.40
Corn, %	18.80
Molasses, %	2.50
Cottonseed meal, %	3.80
Soybean hull, %	4.40
Corn gluten meal, %	6.30
Corn husk, %	12.50
Expanded urea, %	0.80
NaCl, %	0.40
Expanded urea, %	0.40
Premix, %	0.30
**Nutritional levels**	
DM, % as fed	91.74
NDF, % as DM	42.17
ADF, % as DM	21.78
EE, % as DM	1.13
CP, % as DM	17.00

### Date and Sample Collection

During the last 4 days of the experiment, the ice water was added around the over flow bottle to terminate fermentation. On days 10 and 11, about 10 ml of fermenter fluid were collected at 0, 3, 6, 9, and 12 h after the morning feed, the ruminal pH was immediately measured with a mobile pH meter (PHB-4, Shanghai Hongyi instrument Limited, Shanghai, China). Then, 5-ml of rumen fluid was preserved with 1 ml of metaphosphoric acid (25% wt/vol) and stored at −20°C for the determination of VFA. On day 12, about 10 ml of ruminal fluid was collected from each fermenter and immediately stored at −80°C to exact bacterial DNA. On day 13, about 20% of solid contents from each nylon bag were frozen at −80°C for the solid phase bacteria DNA extraction. The bag from each vessel on days 10, 11, and 12 was collected, washed one time with 100 ml of artificial saliva, washed with cold water until the outflow was clear, and stored to determine dry matter (DM) disappearance. The DM disappearance was calculated from the loss in weight after oven drying at 65°C for 48 h by using the following equation: DM disappearance (%) = {(g Sample DM – g Residue DM – g Bag DM)/g Sample DM} ^*^100, and the residues were analyzed for DM, organic matter (OM), neutral detergent fiber (NDF), acid detergent fiber (ADF), and crude protein (CP).

### Analytical Procedures

The content of DM, ash, and N in the feed and residues were determined according to the Association of Official Analytical Chemists (AOAC) method (18). The DM content was determined by drying at 105°C in a forced-air oven for 4 h. The ash content was determined by complete combustion in a muffle furnace (PrepASH-340, Precisa, Swizerland) at 550°C for 6 h. The N contents of the feed bag were carried out by a protein analyzer (K9840, Hanon Advanced Technology Group Co., Ltd, Jinan, China) according to the Kjeldahl method and CP was calculated as N × 6.25 (18). The NDF and ADF were determined by the method of Van Soest et al. (19).

The thawed rumen fluid samples were centrifuged at 2,500 ×g at 4°C for 5 min, and the supernatants were processed as described by Liang et al. (20). The VFA concentrations were measured with gas chromatography (GC) on a Thermo Fisher Trace 1300 GC system (TRACE 1300, Thermo Scientific, Milan, Italy) as described by Li et al. (15). The GC was fitted with a silica capillary column (DB-FFAP, 30 m × 0.32 mm × 0.25 μm, Agilent Technologies Co., Ltd, Santa Clara, CA, USA), and crotonic acid (1% wt/vol) was used as the internal standard. The injector and detector temperatures were set at 240°C. The following temperature program was used: the temperature was increased from 50 to 190°C at a rate of 25°C/min, and the temperature increased was increased to 200°C at 10°C/min for 5 min. Finally, the temperature was increased to 220°C at a rate of 10°C/min and was held for 5 min. The concentration of lactate was determined by a commercial Lactate Analysis Kit (Nanjing Jiancheng Technology Co., LTD., Nanjing, China).

### Microbial DNA Extraction and Relative Quantitative Real-Time PCR

The DNA of rumen bacteria was extracted by an E.Z.N. A® Bacterial DNA Kit (Omega Bio-Tek, Inc., Norcross, GA, USA) according to the instructions from the manufacturer. The final elution volume was 80 μl, and DNA concentration and purity were measured by an ND-2000 spectrophotometer (NanoDrop Technologies, Wilmington, DE, USA). The primer design for all the rumen bacteria to amplify was selected on the basis of the published literature (Table 3). The quantitative real-time PCR (qPCR) protocol was described by Liang et al. (20). Each sample contained 1 μl of DNA, 10 μl of SYBR Green (TransGen Biotech, Beijing, China), 0.6 μl of each primer, and 8.6 μl of ddH_2_O in a final volume of 20 μl. The amplification conditions were as follows: 95°C for 10 s; 40 cycles of 10 s at 95°C; 30 s at 60°C; 72°C for 10 s; and a final cycle at 72°C for 5 min. To obtain melting curve data, the temperature increased in 0.5°C increments from 65 to 95°C. All investigated PCR products had only single melting peaks. The relative abundance of rumen bacteria was expressed as a proportion of total rumen bacterial 16S rRNA according to the equation: relative quantification = 2^−(*CTctarget*−*CTctotal bacteria*)^, where *CT* represents a threshold cycle (26). Before the statistical analysis, the percentage of each microbe target was calculated as (2^−Δ*CT*^) × 100, then, the data were log10–scale transformed before the statistical analysis (20). The quantity of each species was expressed as the log10 copy number of 16S rRNA gene copies per milliliter of rumen fluid.

**Table 3:** The sequence of primers used to analyze the relative abundance of bacteria.

**Primer name**	**Primer sequences (5′–3′)**	**References**
*Fibrobacter succinogenes*	F: 5-GGTATGGGATGAGCTTGC-3 R: 5-GCCTGCCCCTGAACTATC-3	(21)
*Butyrivibrio fibrisolvens*	F: 5-GCCTCAGCGTCAGTAATCG-3 R: 5-GGAGCGTAGGCGGTTTTAC-3	(22)
*Ruminococcus flavefaciens*	F: 5-CGAACGGAGATAATTTGAGTTTACTTAGG-3 R: 5-CGGTCTCTGTATGTTATGAGGTATTACC-3	(22)
*Prevotella brevis*	F: 5-GGTTCTGAGAGGAAGGTCCCC-3 R: 5-TCCTGCACGCTACTTGGCTG-3	(23)
*Selenomonas ruminantium*	F: 5-CAATAAGCATTCCGCCTGGG-3 R: 5-TTCACTCAATGTCAAGCCCTGG-3	(23)
*Ruminococcus albus*	F: 5-CCCTAAAAGCAGTCTTAGTTCG-3 R: 5-CCTCCTTGCGGTTAGAAC-3	(24)
*Total bacteria*	F: 5-TCCTACGGGAGGCAGCAGT-3 R: 5-GGACTACCAGGGTATCTAATCCTGTT-3	(25)

The sequence analysis and bioinformatics were conducted by SMRT Portal (version. 2.7; PacBio, CA, USA). The Lima (version. 1.7.0; PacBio, CA, USA) software was applied to export circular consensus sequencing (CCS) sequences from raw data and perform Barcode identification for the CCS sequences. Then, the chimera was filtered by UCHIME (version. 4.2; Tiburon, CA, USA) software to get the Optimization-CCS (27). We cluster Optimization-CCS sequences to get operational taxonomic units (OTU) by USEARCH (version 10.0; Tiburon, CA, USA) software (28), then get the species classification according to the sequence composition of OTU. The principal coordinate analysis (PCoA) plot of samples according to the distance matrix was obtained to analyze. The Ace, Chao1, Shannon, and Simpson indexes of each sample were statistically calculated by using Mothur (version v.1.30; Mothur, Michigan, USA) to evaluate the alpha diversity at 97% similarity level (29). According to OTU analysis results, a taxonomic analysis was performed with RDP Classifier (version 2.2; RDP Classifier, Michigan, USA) at the taxonomic level of phylum and genus (30). The raw sequencing data were in the Sequence Read Archive (SRA) of NCBI and can be accessed *via* accession number: PRJNA752826.

### Statistical Analysis

The nutrients degradation, fermentation parameters, and rumen bacteria abundances were analyzed by using SPSS software version 17.0 (IBM, Armonk, NY, United States). The independent sample *T*-test was used to calculate the differences in the results between the high AS pH group and the low AS pH group in this experiment. The effect of time on fermentation variables was used as a repeated measure. The model included the effects of AS pH, time, and their interaction as fixed effects, and individual fermenters as a random effect. The Kruskal–Wallis test was used to test the rumen bacteria in the solid and liquid fraction at the phylum and genus. The significant difference of data was analyzed by Kruskal–Wallis one-way ANOVA analysis. The significance was set as *P* ≤ 0.05 and the tendencies were considered when 0.05 < *P* < 0.10.

## Results

Decreasing the AS buffer capacity resulted in a reduction in average pH to 6.02 in the low AS group. The effect of AS pH on the nutrients degradabilities is presented in Table 4. The degradabilities of DM, OM, NDF, ADF, and CP were lower in the low AS pH group compared with the high AS pH group (*P* < 0.001).

**Table 4:** Effect of AS pH on the nutrients degradability in the rumen simulation technique (RUSITEC).

**Degradability rate, % DM**	**High AS pH**	**Low AS pH**	**SEM^***a***^**	***P*-value**
DM	64.37 ± 0.72	58.67 ± 1.37	0.555	<0.001
OM	64.38 ± 1.26	59.32 ± 1.34	0.669	<0.001
NDF	46.87 ± 0.83	39.94 ± 2.10	1.001	<0.001
ADF	38.16 ± 1.54	31.13 ± 2.51	1.274	<0.001
CP	70.33 ± 1.76	62.99 ± 2.83	1.239	<0.001

*SEM^a^, standard error of the sample means*.

The effect of AS pH on VFA in RUSITEC is shown in Table 5. The total concentration of VFA tended to be lower (*P* = 0.080) in the low AS pH group compared with the high AS pH group. The proportions of acetate, isobutyrate, and the concentration of lactate were not affected by different AS pH (*P* > 0.05). The low AS pH decreased the proportion of propionate (*P* < 0.001) and increased the proportions of butyrate (*P* = 0.008), isovalerate (*P* = 0.012), valerate (*P* < 0.001), and the ratio of acetate to propionate (*P* < 0.001).

**Table 5:** Effect of artificial saliva (AS) pH on pH and volatile fatty acids in the rumen simulating fermenter (RUSITEC).

	**High AS pH**	**Low AS pH**	**SEM^***a***^**	***P*-value**
pH	7.03 ± 0.05	6.02 ± 0.05	0.023	<0.001
**VFA molar ratios, mol/100 mol**				
Acetate	46.47 ± 0.74	45.24 ± 1.25	0.727	0.140
Propionate	35.84 ± 1.59	24.94 ± 1.88	1.232	<0.001
Isobutyrate	0.04 ± 0.002	0.04 ± 0.003	0.002	0.161
Butyrate	8.57 ± 1.87	14.18 ± 0.32	0.951	0.008
Isovalerate	1.68 ± 0.33	5.50 ± 2.13	1.075	0.012
Valerate	7.39 ± 0.60	10.07 ± 0.41	0.363	<0.001
Acetate:propionate	1.30 ± 0.04	1.82 ± 0.14	0.723	<0.001
Lactate, mmol/L	0.07 ± 0.01	0.05 ± 0.02	0.010	0.209
TVFA^b^, mmol/L	52.41 ± 8.77	42.66 ± 2.92	4.624	0.080

*SEM^a^, standard error of the sample means; TVFA^b^, total volatile fatty acids*.

The effect of AS pH on the fermentation parameters at 0, 3, 6, 9, and 12 h after feeding is shown in Supplementary Table 1. An interaction between AS pH and time affected the rumen pH (*P* = 0.003); the low AS pH had lower rumen pH than high AS pH (*P* < 0.001), and rumen pH was decreased at 0 and 9 h after feeding (*P* < 0.001). The proportion of acetate was increased at 0 h and decreased at 3 h after feeding in the high AS pH group, respectively (*P* = 0.039). The proportion of butyrate was affected by an interaction between the AS pH and time (*P* = 0.037). The low AS pH had a greater proportion of butyrate than high AS pH (*P* = 0.002), and the proportion of butyrate was decreased at 0 h and increased at 3 and 9 h between the low and high AS pH group (*P* < 0.001). The proportion of valerate was higher at 0, 3, 6, and 9 h than at 12 h after feeding in the low AS pH group (*P* = 0.009).

The effect of AS pH on the α diversity and phylum abundances of the ruminal bacteria are shown in Table 6. The sequence coverage sufficiently met a coverage >97% for all the samples. Across all the samples, a total of 102,043 CCS sequences were obtained, and an average of 6,378 CCS sequences per sample. In total, 94% of CCS sequences were classified at the phyla level and 66% at the genus level. In the solid-associated bacteria, the ACE, Chao1, and Simpson indexes were not affected by AS pH (*P* > 0.05). However, the Shannon index was greater in the high AS pH group (*P* = 0.031) compared with the low AS pH group. In the liquid-associated bacteria, the ACE, Chao1, and Shannon indexes were greater (*P* < 0.05) in the high AS pH group compared with the low AS pH group, whereas the Simpson index tended to be higher (*P* = 0.098). The ACE, Chao1, Simpson, and Shannon indexes have no difference between solid-associated bacteria and liquid-associated bacteria.

**Table 6:** Effect of AS pH on the α-diversity of the rumen bacteria and community at phylum level in RUSITEC.

		**High AS pH**	**Low AS pH**	**SEM^**1**^**	***P*** **-value**	
					**AS pH**	**Rumen bacteria**
**α-diversity**						
ACE	Solid	168.34 ± 25.19	190.62 ± 25.60	17.956	0.261	0.075
	Liquid	219.64 ± 12.85	179.92 ± 15.22	9.961	0.007	
Chao1	Solid	162.49 ± 25.37^ab^	184.05 ± 16.48^b^	16.126	0.204	0.030
	Liquid	218.33 ± 9.27^a^	183.69 ± 24.38^ab^	13.021	0.038	
Simpson	Solid	0.09 ± 0.22^ab^	0.13 ± 0.04^a^	0.227	0.126	0.009
	Liquid	0.03 ± 0.00^b^	0.12 ± 0.08^ab^	0.038	0.098	
Shannon	Solid	3.38 ± 0.25^ab^	2.93 ± 0.19^b^	0.157	0.031	0.008
	Liquid	4.22 ± 0.12^a^	3.22 ± 0.46^ab^	0.235	0.006	
**Phylum, %**						
*Firmicutes*	Solid	44.65 ± 2.07	47.35 ± 8.87	4.552	0.574	0.075
	Liquid	32.94 ± 1.83	31.02 ± 12.50	6.317	0.373	
*Bacteroidetes*	Solid	47.50 ± 2.63^a^	39.68 ± 9.56^ab^	4.957	0.166	0.030
	Liquid	37.60 ± 1.49^ab^	29.74 ± 8.17^b^	4.150	0.107	
*Proteobacteria*	Solid	5.26 ± 1.94^b^	7.87 ± 4.01^ab^	2.229	0.286	0.009
	Liquid	12.82 ± 3.43^ab^	27.53 ± 5.72^a^	3.335	0.005	
*Planctomycetes*	Solid	0.88 ± 0.80^ab^	0.37 ± 0.48^b^	0.465	0.313	0.008
	Liquid	10.70 ± 2.43^a^	2.26 ± 1.07^ab^	1.328	0.001	
*Spirochaetes*	Solid	0.88 ± 0.34^ab^	3.15 ± 0.66^a^	0.371	0.001	0.005
	Liquid	1.35 ± 0.49^ab^	0.06 ± 0.048^b^	0.245	0.002	
*Verrucomicrobia*	Solid	1.67 ± 0.72^a^	0.06 ± 0.06^ab^	0.023	0.004	0.082
	Liquid	0.02 ± 0.04^b^	0.006 ± 0.01^b^	0.361	0.510	
*Actinobacteria*	Solid	0.02 ± 0.03	0.95 ± 1.21	0.603	0.221	0.105
	Liquid	0.01 ± 0.02	0	0.012	0.391	
*Tenericutes*	Solid	0.20 ± 0.13	0.33 ± 0.23	0.132	0.386	0.364
	Liquid	0.31 ± 0.12	0.06 ± 0.07	0.071	0.012	
*Lentisphaerae*	Solid	0.03 ± 0.03^ab^	0^b^	0.016	0.190	0.040
	Liquid	0.40 ± 0.20^a^	0.03 ± 0.02^ab^	0.099	0.031	
*Synergistetes*	Solid	0.006 ± 0.01^b^	0.05 ± 0.02^ab^	0.010	0.004	0.060
	Liquid	0.15 ± 0.10^ab^	0.25 ± 0.17^a^	0.100	0.342	
*Others*	Solid	0.01 ± 0.02^ab^	0^b^	0.011	0.391	0.311
	Liquid	0.13 ± 0.12^ab^	0.17 ± 0.08^a^	0.073	0.586	
*Unclassified*	Solid	0.54 ± 0.29^ab^	0.25 ± 0.14^b^	0.162	0.134	0.006
	Liquid	1.92 ± 0.54^a^	0.82 ± 0.12^ab^	0.275	0.024	

a,b *Differences (P < 0.05) between the abundance of rumen bacteria within solid fraction and liquid fraction*.

*SEM^1^, standard error of the sample means*.

The abundance of *Firmicutes* and *Bacteroidetes* was not affected by AS pH treatment (*P* > 0.05). The low AS pH increased the abundances of *Spirochaetes* (*P* = 0.001), *Synergistetes* (*P* = 0.004), and decreased the *Verrucomicrobia* abundance (*P* = 0.004) in the solid-associated bacteria. In the liquid-associated bacteria, low AS pH increased the abundance of *Proteobacteria* (*P* = 0.005) and decreased the abundances of *Planctomycetes* (*P* = 0.001), *Spirochaetes* (*P* = 0.002), *Tenericutes* (*P* = 0.012), and *Lentisphaerae* (*P* = 0.031). At the AS pH 6.0, the abundance of *Spirochaetes* was greater in solid than a liquid fraction. For the liquid and solid fraction, the abundances of *Firmicutes, Bacteroidetes*, and *Proteobacteria* were similar in solid fraction compared with the liquid fraction.

**Figure 1:**
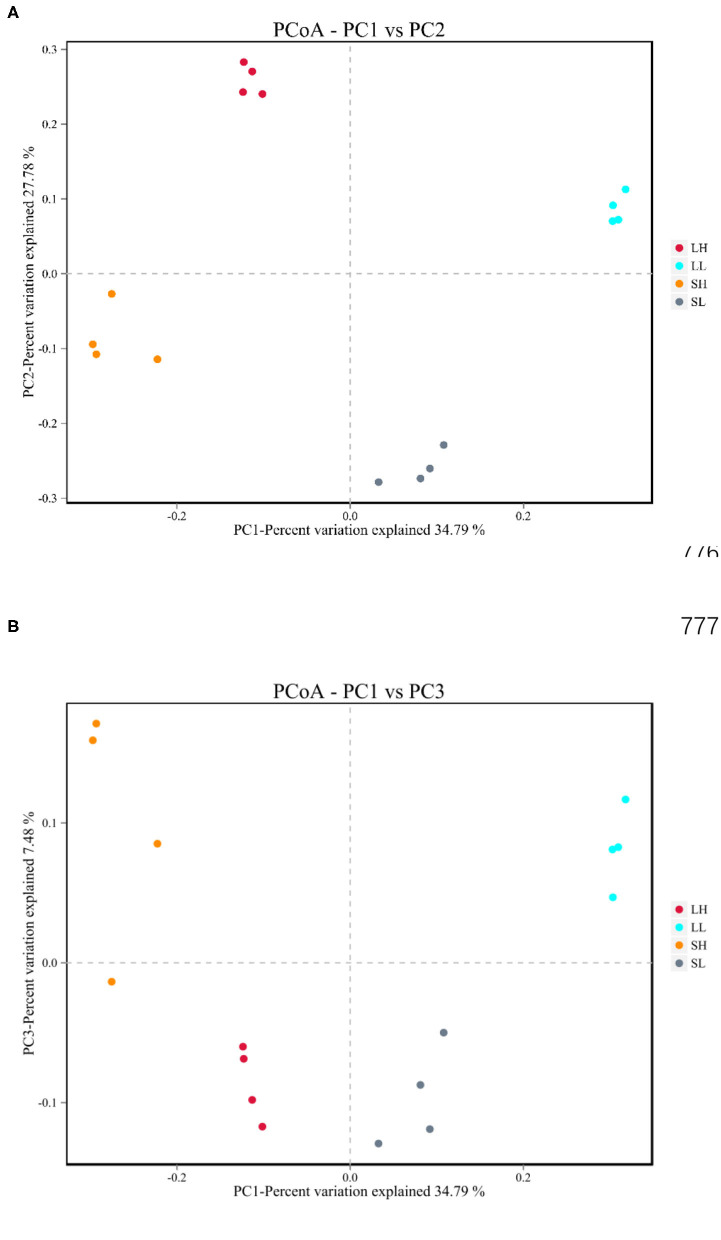
Effects of artificial saliva (AS) pH on the β diversity in the rumen bacteria **(A,B)** in RUSITEC. LH, the high artificial saliva (AS) pH in the liquid-associated bacteria; LL, the low AS pH in the liquid-associated bacteria; SH, the high AS pH in the solid-associated bacteria; SL, the low AS pH in the solid-associated bacteria.

The beta diversities of bacteria communities within different AS pH for each fraction were calculated and visualized through the two-dimensional PCoA analysis using the binary-Jaccard (Figure 1). A significant difference between the bacterial communities in the AS pH treatment was noted. Both principal components accounted for 34.79% (PC1) and 27.78% (PC2) of the explained variance.

The ruminal bacteria with abundances >1% at the genus level were presented in Table 7. The low AS pH increased the abundance of *Lactobacillus* (*P* = 0.050) and decreased the abundances of *Schwartzia* (*P* = 0.002) in solid-associated bacteria compared with the high AS pH group. In the solid-associated bacteria, the abundance of *Succinivibrio* tended to be greater (*P* = 0.059) in the low AS pH group compared with the high AS pH group, whereas the abundance of *Ruminobacter* tended to be lower (*P* = 0.086). In the liquid-associated bacteria, the abundances of *Prevotellaceae_YAB2003_group* (*P* = 0.040), *Schwartzia* (*P* = 0.002), and *Ruminobacter* (*P* = 0.043) were lower in the low AS pH group compared with the high AS pH group. However, the abundances of *Succinivibrio* (*P* < 0.001) and *Prevotella_1* (*P* = 0.001) were higher in the low AS pH treatment compared with the high AS pH group. At the AS pH 7.0 and 6.0, the abundance of *Prevotellaceae_YAB2003_group* was greater in a solid fraction than a liquid fraction (*P* < 0.05). The abundance of *Prevotella_1* was decreased in a solid fraction when the AS pH was 6.0 (*P* = 0.008), while the abundance of *Prevotella_7* was increased (*P* = 0.007).

**Table 7:** Effect of AS pH on the rumen bacteria at genus level in RUSITEC.

		**High AS pH**	**Low AS pH**	**SEM^**1**^**	***P*** **-value**	
					**AS pH**	**Rumen bacteria**
*Lactobacillus*	Solid	17.35 ± 5.96^ab^	29.32 ± 7.76^a^	4.895	0.050	0.019
	Liquid	4.55 ± 1.20^b^	19.21 ± 18.54^ab^	9.292	0.212	
*Prevotellaceae_YAB2003_group*	Solid	25.91 ± 4.41^a^	23.91 ± 11.57^a^	6.129	0.755	0.005
	Liquid	7.51 ± 3.99^b^	0.64 ± 0.53^b^	2.014	0.040	
*Succinivibrio*	Solid	3.90 ± 1.51^b^	7.47 ± 4.12^ab^	2.193	0.059	0.012
	Liquid	6.28 ± 2.14^ab^	25.59 ± 5.24^a^	2.831	<0.001	
*Selenomonas_1*	Solid	6.20 ± 1.160	7.85 ± 2.18	1.234	0.206	0.481
	Liquid	7.81 ± 1.29	8.99 ± 3.84	2.025	0.592	
*Prevotella_1*	Solid	6.14 ± 1.45^ab^	3.38 ± 1.42^b^	1.017	0.340	0.008
	Liquid	3.05 ± 0.71^b^	14.09 ± 3.61^a^	1.841	0.001	
*Rikenellaceae_RC9_gut_group*	Solid	2.96 ± 1.09^b^	3.16 ± 1.43^ab^	0.898	0.827	0.009
	Liquid	8.68 ± 2.59^ab^	10.92 ± 3.88^a^	2.330	0.374	
*Prevotella_7*	Solid	4.30 ± 2.19^ab^	6.89 ± 2.07^a^	1.507	0.137	0.007
	Liquid	1.76 ± 0.90^ab^	0.38 ± 0.12^b^	0.454	0.053	
*Schwartzia*	Solid	5.20 ± 0.98^a^	2.34 ± 0.38^ab^	0.523	0.002	0.005
	Liquid	2.13 ± 0.25^ab^	1.30 ± 0.39^b^	0.229	0.011	
*Ruminobacter*	Solid	1.00 ± 0.78^ab^	0.15 ± 0.02^b^	0.390	0.086	0.005
	Liquid	4.66 ± 2.76^a^	0.01 ± 0.01^ab^	1.381	0.043	
*Succiniclasticum*	Solid	0.34 ± 0.27^b^	0.57 ± 0.40^ab^	0.243	0.391	0.012
	Liquid	1.70 ± 0.77^ab^	3.01 ± 1.50^a^	0.840	0.171	
*Others*	Solid	18.39 ± 4.59^ab^	11.35 ± 2.71^ab^	2.651	0.038	0.013
	Liquid	21.43 ± 3.36^a^	10.18 ± 3.59^b^	2.459	0.004	
*Unclassified*	Solid	8.38 ± 4.25^b^	3.75 ± 0.86^ab^	2.168	0.076	0.009
	Liquid	3.04 ± 3.21^a^	5.67 ± 1.10^ab^	1.695	<0.001	

a,b *Differences (P < 0.05) between the abundance of rumen bacteria within the solid fraction and liquid fraction*.

*SEM^1^, standard error of the sample means*.

The effect of AS pH on the number of rumen bacteria is presented in Table 8. The low AS pH decreased the number of *Ruminococcus albus, R. flavefaciens, F. succinogenes* (*P* < 0.001) both in the solid- and liquid-associated bacteria, respectively. The low AS pH tended to increase the amount of *Prevotella brevis* (*P* = 0.091) in liquid-associated bacteria. The low AS pH decreased the amounts of *Selenomonas ruminantium* in solid-associated bacteria (*P* = 0.022) and tended to decrease in liquid-associated bacteria (*P* = 0.065). The number of *S. ruminantium* was increased in solid fractions both in high AS pH and low AS pH (*P* < 0.001). At the high AS pH, the amounts of *P. brevis, Butyrivibrio fibrisolvens*, and *total bacteria* were increased in solid fraction compared with the liquid fraction (*P* < 0.05). The number of *F. succinogenes* was greater in solid fraction than liquid fraction at the low AS pH (*P* < 0.001).

**Table 8:** Effect of AS pH on the number of ruminal bacteria in RUSITEC.

		**High AS pH**	**Low AS pH**	**SEM^**1**^**	***P*** **-value**	
					**AS pH**	**Rumen bacteria**
*Ruminococcus Flavefaciens*	Solid	9.85 ± 0.89^a^	7.24 ± 0.98^b^	0.399	<0.001	<0.001
	Liquid	9.19 ± 0.21^a^	7.05 ± 0.47^b^	0.232	<0.001	
*Fibrobacter succinogenes*	Solid	8.59 ± 0.67^a^	7.86 ± 0.31^a^	0.223	<0.001	<0.001
	Liquid	7.75 ± 0.22^a^	6.36 ± 0.45^b^	0.192	<0.001	
*Prevotella brevis*	Solid	9.38 ± 0.24^a^	9.32 ± 0.45^a^	0.148	0.693	0.001
	Liquid	8.85 ± 0.42^b^	9.09 ± 0.18^ab^	0.132	0.091	
*Ruminococcus albus*	Solid	10.22 ± 0.54^a^	8.26 ± 0.22^b^	0.254	<0.001	<0.001
	Liquid	9.69 ± 0.21^a^	7.77 ± 0.41^b^	0.213	<0.001	
*Selenomonas ruminantium*	Solid	10.78 ± 0.24^a^	10.50 ± 0.29^a^	0.453	0.028	<0.001
	Liquid	10.26 ± 0.16^b^	10.14 ± 0.13^b^	0.121	0.065	
*Butyrivibrio fibrisolvens*	Solid	8.94 ± 0.40^a^	8.54 ± 0.28^ab^	0.145	0.081	0.011
	Liquid	8.40 ± 0.10^b^	8.55 ± 0.18^ab^	0.058	0.022	
*Total bacteria*	Solid	15.59 ± 0.49^a^	15.55 ± 0.40^a^	0.178	0.833	0.002
	Liquid	15.03 ± 0.18^b^	15.34 ± 0.15^ab^	0.072	<0.001	

a,b *Differences (P < 0.05) between the number of rumen bacteria within the solid fraction and liquid fraction*.

*SEM^1^, standard error of the sample means*.

## Discussion

The rumen pH is the most monitored parameter for SARA diagnosis. According to the severity of SARA, the average daily pH threshold was 5.50–6.25 (3). In the current study, decreasing the AS buffer capacity resulted in an average pH of 6.02. The ruminal pH was an important factor that affect the degradation of NDF and OM degradation in the rumen (31). In our study, low AS pH decreased the degradabilities of DM, OM, NDF, and ADF. These results are consistent with the previous reports that the digestion rates of DM and NDF were reduced with the decreasing AS pH (7.0 vs. 4.9) *in vitro* (32). The decreased digestibilties of NDF and ADF at the low AS pH are mainly attributed to the reduction of cellulolytic bacteria populations and the ability of cellulolytic bacteria to attach to the feed particles (33).

Reduction in the rumen pH lower than 6.0 has a negative impact on the amount of cellulolytic bacteria (*R. albus, R. R. flavefaciens, F. succinogenes*, and *B. fibrisolvens*) in the rumen (15, 34). As expected, the low AS pH decreased the amount of ruminal *R. albus, R. flavefaciens, F. succinogenes*, and *B. fibrisolvens* in the solid- and liquid-associated bacteria with the identical substrates chemical compositions. However, Li et al. (8) found that the ruminal cellulolytic bacteria, such as the amounts of *F. succinogenes* and *R. flavefaciens* were increased when the dairy goats experience the low-peNDF diet induced SARA. Khafipour et al. (6) found the mild grain-induced SARA increased the populations of *R. albus* and *R. flavefaciens*. The result of cellulolytic bacteria was not consistent between the low peNDF-induced SARA with the high grained-induced SARA *in vivo* (6). In the present study, we intended to stimulate the low peNDF induced SARA by decreasing the AS pH in Rustitec, which is similar to the decreased saliva secretion when the ruminants received the low peNDF diets *in vivo*. The increased cellulolytic bacteria when the cows or goats received the low peNDF diets (decreased the roughage particle size without changing the roughage to concentrate ratio) mainly attributed to the increased surface area for microbial attachment (8, 35–37). However, the particle size of feed was identical between the treatments, and the changes of cellulolytic bacteria only response to the different AS pH in the present study. Therefore, the results of the present study indicated that the low AS pH indeed decreased the number of cellulolytic bacteria when the substrate was identical in the Rustitec. The rumen bacteria abundance of the solid fraction is significantly higher than that of the liquid fraction (38). The amounts of *B. fibrisolvens* and *F. succinogenes, total bacteria* were enriched in a solid fraction in our study. This result was in accordance with De Mulder et al. (14), who identified that cellulolytic bacteria are prevalent in the solid fraction.

The ruminal genus of *Prevotella.app* is considered to be associated with starch degradation and growth well at low pH conditions (6, 39). In our study, the amount of *P. brevis* was not affected by AS pH in the solid-associated bacteria, but the number of *P. brevis* in the liquid-associated bacteria tended to be increased in the low AS pH group.

In the current study, the degradability of CP decreased in the low AS pH group. Several studies have indicated that the low AS pH decreased or unaffected CP degradability (32, 40). The plant proteins were integrated within non-protein polymers, such as polysaccharides, which may limit the access of proteolytic bacteria to the substrate (41). It is possible that the low AS pH decreased ruminal cellulolytic activity and led to a reduction in the CP degradability due to the limitation of the access of proteases to their matrix (8).

In our experiment, the total concentration of VFA was reduced in the low AS pH treatment. This result was in accordance with Jiao et al. (42), who found that the total concentration of VFA (42.66 vs. 52.41 mmol/L) declined when pH was at 5.8 compared with the pH 6.5 *in vitro*. The declined total VFA concentration in the present study is mainly attributed to the decreased OM degradability. In our study, the proportion of acetate was increased at 0 h and decreased at 3 h after feeding in the high AS pH group. Because the fermenters are opened to supply new nylon bags with feed; this operation exposes the cellulolytic bacteria to oxygen and inhibits the activity (43). The decrease AS pH reduced the proportion of propionate in our experiment. The results were consistent with Strobel and Russell (44) found the concentration of propionate from starch fermentation (2.9 vs. 1.1 mM) decreased when the pH decreased from 6.7 to 5.8. The previous studies reported the amylolytic bacteria to produce amounts of propionate, but many cellulolytic bacteria generate a large amount of succinate, an intermediate that is eventually converted to propionate (45). The decreased proportion of propionate was because the low ruminal pH inhibited the succinate conversion to propionate. The lower molar ratio of the propionate in the low AS pH group also resulted in a higher acetate to propionate compared with the high AS pH group. These results are different from Cardozo et al. (46), who reported the ratio of acetate to propionate was lower when pH was decreased from 7.0 to 5.5 because the high-grain diets decreased the acetate production and increased the propionate production in the rumen.

In the present study, the proportion of butyrate was greater in the low AS pH group compared with the high AS pH group. The results were in accordance with Esdale and Satter (47), who reported that the butyrate production was higher at pH 5.6 compared with at pH 6.2 *in vitro*. In addition, Shriver et al. (48) found butyrate production increased as pH was decreased from 6.2 to 5.8 *in vivo*. Calsamiglia et al. (32) identified that the concentration of butyrate was only affected by the changes of pH and not affected by diet compositions *in vitro*. The results could be associated with the increasing abundance of *Prevotellaceae* (e.g., *Prevotella_1*) in the liquid-associated bacteria that resulted in the increased butyrate production *in vitro* (49). In the current study, the proportion of butyrate was decreased at 0 h and increased at 3, 6, and 9 h after feeding. At 2 h after the start of incubation, the16S rDNA copy numbers of amylolytic bacteria attached to the grain were increased (50), which may promote butyrate production at 3, 6, and 9 h after feeding.

The concentrations of isovalerate and valerate in the rumen were related to the protein degradation and fermentation of branched-chain AA (51). The isovalerate and valerate are also considered as stimulating factors that enhanced the growth of cellulolytic bacteria (52). In the current study, the low AS pH increased the proportion of isovalerate and valerate, and the proportion of valerate was higher at 0, 3, 6, and 9 h than at 12 h after feeding. It had a low pH, which inhibited the growth of cellulolytic bacteria. The RUSITEC system fermenters were opened when the bags were replaced by the new nylon bags with feed; this operation exposed cellulolytic bacteria to oxygen and inhibited the activity of cellulolytic bacteria (43). This therefore would have resulted in the accumulation of valerate and isovalerate in the fermenters and increased at 3, 6, 9, and 12 h after feeding.

Using the sequence and bioinformatics analysis, we obtained 6,378 CCS sequences on average for each sample with good coverage (>97.0%). In accordance with our hypothesis, both the microbial α-diversity and β-diversity were affected by AS pH treatment. Meanwhile, most of the alpha diversity indices (except Simpson index) decreased with the low AS pH in the liquid-associated bacteria, suggesting that the low pH significantly decreased the activity and number of ruminal bacteria. These results are in agreement with Shen et al. (53), who reported the reduction of pH decreased the bacteria alpha diversity. In addition, the PCoA analysis also showed that the bacterial communities of the high AS pH and low AS pH clustered separately, indicating their distinct bacterial compositions in the rumen. These results were similar to the founding by Li et al. (15) who found the bacterial compositions were different between the sheep with high rumen pH and low rumen pH with identical feed composition. Interestingly, the ACE, Chao1, Simpson, and Shannon indexes have no difference between solid fractions and liquid fractions in our study. Because the solid- and liquid-associated bacteria do not have differences in the taxonomic composition but can be distinguished based on the relative abundance of species (14).

In the present study, the relative abundances of ruminal *Firmicutes* and *Bacteroidetes* were not affected by AS pH. The *Firmicutes* are predominantly composed of Gram-positive bacteria in the rumen, which are metabolically capable of utilizing the fermentable carbohydrates (54). Previous studies showed that feeding high-grain diets for cattle increased the abundance of ruminal *Firmicutes* (55). However, AS pH did not affect the abundance of *Firmicutes* in our study suggests that the *Firmicutes* were pH-insensitive bacteria. The *Bacteroidetes* are the most abundant Gram-negative bacteria found in the anaerobic communities of the rumen, and low pH resulted in the death and lysis of Gram-negative bacteria (15, 54). However, the abundance of *Bacteroidetes* was not affected by AS pH in the present study. Although the *Bacteroidetes* were not different in statistics, the value of *Bacteroidetes* decreased in the low pH group (47.50 vs. 39.68% in solid-associated bacteria and 37.60 vs. 29.74% in liquid-associated bacteria). Wang et al. (56) reported that feeding high-concentrate diets decreased the ruminal pH and increased the abundance of *Proteobacteria* in the rumen for cows. Furthermore, the low AS pH increased the abundance of *Proteobacteria* in the liquid-associated bacteria in this experiment. This result suggests that the phylum of *Proteobacteria* can tolerate the low pH condition. For the liquid and solid fraction, the abundances of *Firmicutes, Bacteroidetes*, and *Proteobacteria* were similar in solid fraction compared with the liquid fraction in our study. It is possible that the fermenters were moved up and down by an electric motor, and promoted the exchange of rumen bacteria in solid fraction and liquid fraction. The *Spirochaetes* commonly fermented xylan and pectin in feed (57). After 6 h fermentation, the *Spirochaetes* phyla became abundant in the forage-adherent community (58). The *Spirochaetes* was greater in low pH conditions in the solid-associated bacteria, which was also greater in high AS pH in the liquid-associated bacteria in the current study. And the abundance of *Spirochaetes* was greater in a solid fraction than a liquid fraction. These results indicated that *Spirochaetes* tend to colonize in the solid phase in the rumen.

The bacteria genus of *Lactobac*ria was suitable for growth at pH 6.0 (59). The previous studies indicated that the increased non-fiber carbohydrate for ruminant promoted the growth of amylolytic and other starch-digesting bacterial species, such as *Lactobacillus* (60–62). In the current study, the relative abundance of *Lactobacillus* was increased when the AS pH decreased. Wang et al. (63) reported that the abundance of ruminal *Lactobacillus* was increased when cows intake the SARA diet. These studies indicated that *Lactobacillus* affected not only the dietary compositions but also the magnitude of pH. The *Prevotella* species were essential to hemicellulose degradation in the rumen, and *Prevotella_1* and *Prevotellaceae_YAB2003* (*Bactenroidetes*) were identified to have the ability to degrade hemicellulose or xylan *in vivo* (64, 65). In the current study, the low AS pH increased the abundances of *Prevotella_1*, and decreased the abundances of *Prevotellaceae_YAB2003*, and *Prevotella_7* in the liquid-associated bacteria. These results indicate the sensitivity of *Prevotella* strains to AS pH was inconsistent. Similarly, the abundance of *Prevotellaceae_YAB2003_group* was greater in a solid fraction than a liquid fraction. The abundance of *Prevotella_1* was decreased in solid fractions at the low AS pH, whereas the abundance of *Prevotella_7* was increased. The *Prevotellaceae* comprises up to 40% of the community in the liquid samples, and ruminal *Prevotella* is non-cellulolytic but has a broad saccharolytic and proteolytic potential (14, 66). The abundances of *Prevotellaceae_YAB2003_group* and *Prevotella_1* were increased in liquid fraction, which primarily consumed the soluble nutrients.

The function of *Succinivibrio* produced succinate, the precursor of propionate (67). In this research, the related abundance of *Succinivibrio* was higher in the low AS pH group compared with low AS pH, whereas the proportion of propionate was decreased. It should be presumed that the conversion of succinate to propionate acid was inhibited by low pH and producing less propionate. In addition, the *Schwartzia* fermented succinate and produced propionate (68). The *Schwartzia* abundance decreased in the low AS pH group, which was coordinated with the results of propionate in this study. The low AS pH decreased the abundance of *Ruminobacter* (*Firmicutes*) in this current study. Wang et al. (69) reported that the cow intake high-forage diets increased the ruminal *Ruminobacter* abundance. Mu et al. (70) found that fed a high grain-diet induce cow SARA increased the abundance of *Ruminobacter*. These results indicated that the growth of *Ruminobacter* in the rumen was affected by the combination of pH and diet compositions.

## Conclusions

The nutrients degradabilities were decreased by reducing AS pH in the present study. The reduction of AS pH increased the proportion of butyrate, valerate, and isovalerate and decreased the proportion of propionate. The results of the present study indicated the three groups of bacteria communities according to the different sensitives to rumen pH: the abundances of *Lactobacillus, Succinivibrio, Prevotella_*7 are increased with decreasing AS pH; the amounts of *R. albus, R. flavefaciens, F. succinogenes* as well as the abundances of *Schwartzia* and *Ruminobacter* decreased with reducing AS pH; the abundances of *Selenomonas_1, Rikenellaceae_RC9_gut_group*, and *Succiniclasticum* were not affected by AS pH in Rustitec. In addition, the effect of the interaction of rumen pH and diets on the rumen bacteria community should be further investigated.

## Data Availability Statement

The data for this study can be found in the NCBI database (https://www.ncbi.nlm.nih.gov/bioproject/PRJNA752826).

## Ethics Statement

The animal study was reviewed and approved by the Biological Studies Animal Care and Use Committee of Gansu Province, China (2005–12). Written informed consent was obtained from the owners for the participation of their animals in this study.

## Author Contributions

ToG collected the sample, analyzed the data, and drafted the manuscript. TaG, YC, LG, FaL, and GY collected the sample. FeL presented the idea of this manuscript, supported the funding, analyzed the conclusions, and revised the manuscript. All authors contributed to the article and approved the submitted version.

## Funding

The research was financially supported by the National Natural Science Foundation of China, grant number (No. 32072754) and the Natural Science Foundation of Gansu Province (20JR5RA299), China.

## Conflict of Interest

The authors declare that the research was conducted in the absence of any commercial or financial relationships that could be construed as a potential conflict of interest.

## Publisher's Note

All claims expressed in this article are solely those of the authors and do not necessarily represent those of their affiliated organizations, or those of the publisher, the editors and the reviewers. Any product that may be evaluated in this article, or claim that may be made by its manufacturer, is not guaranteed or endorsed by the publisher.
