# Supplementary material for: Changes in the Fermentation and Bacterial Community by Artificial Saliva pH in RUSITEC System
Source: Front Nutr. 2021 Nov 16;8:760316. doi: 10.3389/fnut.2021.760316 (PMC8637203; doi:10.3389/fnut.2021.760316)
Supplement: Supplementary file 1 [file Table_1.docx]

Supplementary Table 1 Effect of AS pH on fermentation parameters at 0, 3, 6, 9, and 12 h after feeding in Rusitec

|  | AS pH | Time | | | | |  |  | *P-value* |  |
| --- | --- | --- | --- | --- | --- | --- | --- | --- | --- | --- |
|  |  | 0 | 3 | 6 | 9 | 12 | SEM^1^ | AS pH | Time | AS pH×Time |
| pH | High AS pH | 6.99^Ab^ | 6.99^Ab^ | 7.21^Aa^ | 6.92^Ab^ | 7.14^Aa^ | 0.032 | <0.001 | <0.001 | 0.003 |
|  | Low AS pH | 5.87^Bd^ | 6.14^Bc^ | 6.56^Ba^ | 6.06^Bc^ | 6.33^Bb^ | 0.058 |  |  |  |
| VFA molar ratios, mol/100 mol | | | | | | | | | | |
| TVFA^2^, mmol/L | High AS pH | 64.86 | 53.49 | 52.56 | 54.88 | 54.27 | 2.457 | 0.236 | 0.500 | 0.860 |
|  | Low AS pH | 54.75 | 44.56 | 52.46 | 46.96 | 48.28 | 2.856 |  |  |  |
| Acetate | High AS pH | 49.46^a^ | 46.70^b^ | 49.13^ab^ | 47.52^ab^ | 47.58^ab^ | 0.385 | 0.072 | 0.019 | 0.971 |
|  | Low AS pH | 51.30 | 48.75 | 51.09 | 48.70 | 48.62 | 0.542 |  |  |  |
| Propionate | High AS pH | 31.66 | 34.63^A^ | 32.85^A^ | 30.72 | 34.29 | 0.766 | 0.002 | 0.070 | 0.345 |
|  | Low AS pH | 27.05 | 24.95^B^ | 25.07^B^ | 24.67 | 30.8 | 0.822 |  |  |  |
| Butyrate | High AS pH | 8.43^b^ | 9.11B^ab^ | 8.84^Bab^ | 10.21^Ba^ | 9.08^ab^ | 0.232 | 0.002 | <0.001 | 0.037 |
|  | Low AS pH | 10.08^b^ | 12.90^Aa^ | 11.07^Ab^ | 12.86^Aa^ | 10.32^b^ | 0.339 |  |  |  |
| Isovalerate | High AS pH | 2.37 | 1.48 | 1.45 | 2.70 | 1.41 | 0.307 | 0.218 | 0.349 | 0.361 |
|  | Low AS pH | 1.83 | 3.48 | 2.97 | 3.57 | 1.88 | 0.359 |  |  |  |
| Valerate | High AS pH | 8.04 | 8.04^B^ | 7.69^B^ | 8.81 | 7.59 | 0.181 | 0.008 | 0.009 | 0.458 |
|  | Low AS pH | 9.74^ab^ | 9.88^Aab^ | 9.76^Aab^ | 10.16^a^ | 8.34^b^ | 0.242 |  |  |  |
| A:P^2^ | High AS pH | 1.59 | 1.35^B^ | 1.50^B^ | 1.60 | 1.39 | 0.500 | 0.003 | 0.104 | 0.475 |
|  | Low AS pH | 1.94 | 1.97^A^ | 2.05^A^ | 1.99 | 1.61 | 0.065 |  |  |  |

SEM^1^: standard error of the sample means. TVFA^2^: total volatile fatty acids.

A: P^2^: acetate: propionate.

^a, b^ Differences (*P* < 0.05) between time points within high AS pH and low AS pH.

^A, B^ Differences (*P* < 0.05) between high AS pH and low AS pH within time points.
